# Predictive and Preventive Mucosal Communications in Particulate Matter Exposure-Linked Renal Distress

**DOI:** 10.3390/jpm11020118

**Published:** 2021-02-11

**Authors:** Yuseok Moon

**Affiliations:** Laboratory of Mucosal Exposome and Biomodulation, Department of Integrative Biomedical Sciences, Biomedical Research Institute, Pusan National University, Yangsan 50612, Korea; moon@pnu.edu; Tel.: +82-51-510-8094

**Keywords:** particulate matter, mucosal exposure, microbiota, chronic kidney disease, mucosal barrier

## Abstract

Despite research into the epidemiological link between exposure to particulate matter (PM) and renal disorder, there is limited information available on the etiological complexity and molecular mechanisms. Among the early responsive tissues to PM exposure, the mucosal barrier of the airway and alimentary tract may be a crucial source of pathologic mediators leading to inflammatory renal diseases, including chronic kidney disease (CKD). Given that harmful responses and products in mucosa exposed to PM may enter the circulation and cause adverse outcomes in the kidney, the aim of the present review was to address the impact of PM exposure on the mucosal barrier and the vicious feedback cycle in the mucosal environment. In addition to the PM-induced alteration of mucosal barrier integrity, the microbial community has a pivotal role in the xenobiotic metabolism and individual susceptibility to PM toxicity. The dysbiosis-induced deleterious metabolites of PM and nutrients are introduced systemically via a disrupted mucosal barrier, contributing to renal injuries and pathologic severity. In contrast, the progress of mucosa-associated renal disease is counteracted by endogenous protective responses in the mucosa. Along with direct elimination of the toxic mediators, modulators of the mucosal microbial community should provide a promising platform for mucosa-based personalized interventions against renal disorders caused by air pollution.

## 1. Introduction

Given the increasing use of fossil fuels and the rapid expansion of desertification, severe air pollution has emerged as an issue of primary concern for human health. Global problems related to airborne particulates have attracted sufficient interest to promote extensive investigation into their adverse effects on biological systems. Particulate matter (PM) is a microscopic aerosol mixture of solid particles and liquid droplets found in the air, derived from natural or anthropogenic origins, including the combustion of fossil fuels and industrial effluents. Anthropogenic PM consists of complex mixtures of mineral oxides, the oxidation products of primary gases, including sulfates, nitrates, and elemental or organic carbon such as volatile organic compounds, and microbial components [1,2,3,4]. Based on the size of the constituent particles, PM is classified into three categories: PM_2.5_ (particles < 2.5 μm in diameter), PM_10_ (particles < 10 μm in diameter), and ultrafine particulates (UFP; particles < 0.1 μm) [5]. The particle size determines the accessibility of particles to inner parts of the mucosal surface in the airway [6].

The adverse health outcomes induced by PM include chronic mucosal inflammatory disorders in the airway and gut, type 2 diabetes (T2D), and cardiovascular and renal dysfunction [7,8,9,10]. Although the ambient particulates may be deposited in the early exposure site, they can translocate through the airway barrier and cause injuries in the vasculature and other target organs via oxidative stress [11,12]. After early exposure to the mucosal tissues of airways or the gut, noxious substances in PM are delivered to the kidney, the organ specialized for filtration and reabsorption, via the circulation [13]. Research regarding the detrimental effects of PM on the kidney has recently been reported, but there is still limited information available on its pathogenic mechanisms [11,14,15,16,17]. However, it is well known that inflammatory and metabolic syndromes, including diabetes and hypertension, are the prevalent causes of CKD [18,19]. Although inflammation and metabolic events can account for the epidemiological evidence supporting a substantial association between exposure to PM and risk of CKD [11,16,17], the mechanistic evidence depicting the harmful effects of PM on the kidneys is not well understood.

Among the stress sentineling tissues, the mucosal barrier is an early producer of mediators of inflammatory and metabolic distress in response to external factors, including microbes and xenobiotics such as PM. The irritated mucosa can transmit stress signals to reprogram a broad range of pathophysiological events. In the present review, it was hypothesized that mucosal responses may influence toxic disorders of the kidney as the ultimate excretion organ. The most distinguishing features of the mucus layer are the main site for nutrient absorption and the microbiota community, which is crucial for regulating complex mucosal communications with the renal pathogenesis and individual susceptibility to environmental factors. Based on the specific early mucosal events, we reviewed the potent etiologies of acute and chronic renal injuries in response to PM exposure, which ultimately suggests the need for personalized interventions based on mucosal communication. 

## 2. Mucosal Etiologies of Renal Injuries Caused by PM

### 2.1. Mucosal Exposure and Translocation of PM

The airway is the primary route of exposure to PM [20]. The penetration of particles is associated with their size, shape, and chemical composition. Generally, PM_10_ can penetrate the deepest parts of the lungs, such as the bronchioles or alveoli. Average exposure to PM_10_ is associated with non-accidental mortality in patients with chronic obstructive pulmonary disease (COPD), especially those diagnosed with asthma-COPD overlap [21]. Moreover, the adverse effects of PM_10_ exposure are relatively severe in women and nonsmokers [21]. Fine particulate matter (PM_2.5_) reaches the gas exchange regions of the lung alveoli, and only nanoscale particles can pass through the air-blood barrier in the acinar region through endocytosis or diffusion and affect other organs via the circulation [22,23,24]. However, larger particles, such as PM_10_ can deposit or be internalized by macrophages, causing detrimental effects on the local tissues and neighboring barrier. Disruption of the barrier would allow translocation of more PM to the circulation and increase the risk to the extrapulmonary organs. 

Particles in inhaled air are cleared by a series of filtration systems. These particles become entrapped in the mucosal layer, and mucociliary transport quickly clears the inhaled PM from the lungs. In the airway regions lacking mucus transport via cilia movement, alveolar macrophages play a crucial role in the defense by phagocytosing foreign particles [25]. The biokinetic fate of inhaled ultrafine radiolabeled particles was examined in rodents [23]. The clearance of an overwhelming proportion of the particles, including ultrafine and micron-sized particles, is mostly mediated by macrophages that transport particles from the peripheral lungs to the larynx, with subsequent passage through the gut and fecal excretion [23]. In addition to gastrointestinal translocation from the airway, PM can enter water and food supply systems directly, and ultimately reach the gastrointestinal tract in humans [26,27]. Each individual ingests approximately 10^12^–10^14^ particles per day (based on a typical Western diet) and the gut mucosa can absorb 1% of ingested PM (10^9^–10^12^ particles per day) given the huge exposure surface of the gut mucosa. In addition to lung exposure, the gastrointestinal tract is another primary deposition site of PM that displays potent early stress responses affecting disease outcomes.

### 2.2. Effects of PM on the Mucosal Barrier

**Figure 1:**
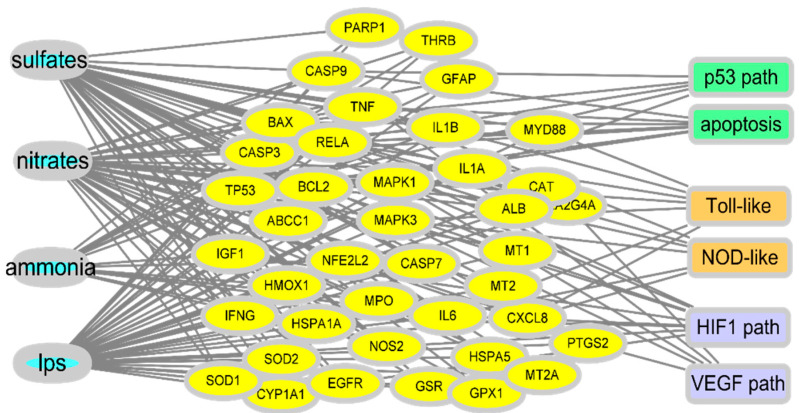
Prediction of gene network in PM-exposed mucosa. Gene expression profiles in the comparative toxicogenomic database in response to oxidation products of the primary gases and endotoxins as a representative microbial product. Based on PM exposure-linked gene sets, Kyoto Encyclopedia of Genes and Genomes (KEGG) analysis was performed to predict events in the insulted tissues.

Extensive parts of the airway and gut linings secrete viscous gel-like substances, known as mucins, as the first line of defense. They are large, highly glycosylated molecules that interact with exogenous substances; this interaction is a critical regulatory step in the migration of particulates to the underlying epithelia through the mucin mesh. Components of PM can directly disrupt this mucosal barrier by lowering the functional efficiency of the mucin structural network. Mechanistically, the collapse phenomenon in the arrangement of mucin networks is speculated to be due to chemical or physical interactions between mucins and external particulate components [28]. The dense connectivity of mucin fiber network loosens as PM-binding increases, with openings for luminal matter, including microbiota, nutrients, and xenobiotic agents. In addition to the impact on the mucosal barrier, PM ingestion can disrupt epithelial permeability [20,29,30]. Permeated PM generates reactive oxygen species (ROS) in epithelial cells [31], which decreases barrier integrity by rearranging or interrupting the epithelial junction in the epithelial lining [32,33]. In vitro evaluation showed reduced transepithelial resistance of the monolayers and structural changes in the tight junctions [20]. The deposition of PM correlated with diminished transcription levels of the tight junction protein 1 and occludin, and with histological evidence of modifications in tight junction organization [20]. Moreover, ingested PM causes gut permeability and aggravates colonic inflammation owing to alterations in cytokine networks [34,35]. In addition to the junctional disruption of the epithelial barrier, PM can cause epithelial cell apoptosis via ROS generation from the mitochondria induced by nuclear transcription factor NF-κB activation [20]. Among the various chemical components of PM, the oxidation products of primary gases are closely associated with a reduction in lifespan expectancy [36]. Conversely, a reduction in sulfate or ammonium is associated with an increase in life expectancy. To examine gene regulation, gene expression profiles were analyzed in cells exposed to PM components, such as sulfate, nitrate, and ammonium as representative life-threatening oxidation products of the primary gases, and endotoxins as a representative microbial product (Figure 1).

In response to exposure to the PM-derived mixture, a network of target genes was revealed. As oxidative radical stress is considered to be the etiology of PM-induced tissue injury, many key network genes are involved in the cell death pathway, including p53. Moreover, another key feature of the network was the association of exposure to inflammatory stress signaling (toll-like receptor and NOD-like receptors) and hypoxia (HIF1 and VEGF pathway). All the molecular associations with cell death, inflammation, and hypoxia signaling pathways indicate the pathological outcomes in the exposed mucosal barrier during PM exposure. Furthermore, the disruption of gut permeability and epithelial injuries is subsequently associated with increased microbial access to the underlying mucosal immune tissues and cells, leading to inflammatory responses and changes in the mucosal microbial community. 

### 2.3. Impact of PM on the Microbial Community

**Figure 2:**
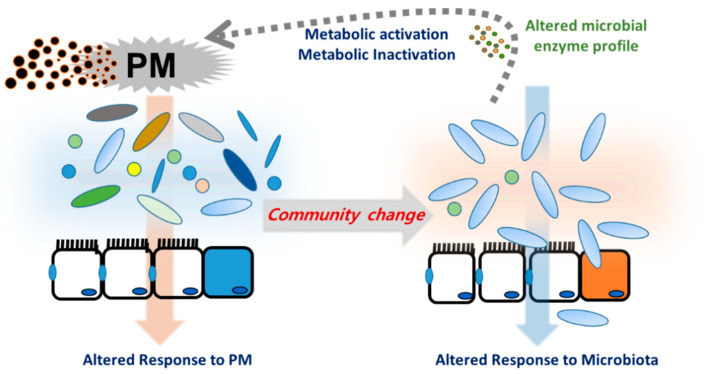
Particulate matter (PM)-induced alternation of mucosal microbial community. Host- and microbiota-derived xenobiotic metabolic enzymes are involved in metabolism of PM constituents. Moreover, PM can directly alter the microbial community, which inversely determines the fate of PM-derived xenobiotics or affect host responses during PM exposure.

The microbiota harbors a complex and dynamic population of microbes, including bacteria, fungi, protozoa, and viruses, which form a continuous microbial community [37] that is built over a lifetime and plays crucial roles in metabolism and immunity in humans and animals [38]. Microbiota are responsive to changes in the luminal environment, such as nutrients and xenobiotic agents. Exposure to PM is thus a key cause of bacterial community changes, which impact immunity and other host physiology. In particular, PM components can induce oxidative stress in gut microbes, leading to the collapse of their community and inducing unexpected health risks in hosts, especially in people with chronic underlying disorders [12]. Long-term exposure to PM_2.5_ may contribute to increased risks of metabolic disorders, including T2D, in humans [39]. Exposure to PM_2.5_ was negatively associated with the alpha diversity index of the gut microbiota, and a lower diversity of the gut microbiota was associated with a higher risk of T2D [39]. In terms of richness, the composition of Firmicutes, Proteobacteria, or Verrucomicrobia phyla was negatively associated with both PM concentrations and the risk of T2D. Moreover, short-term exposure to PM resulted in a dose-dependent reduction in alpha diversity indices of microbiota in the nasal tract, an early mucosal exposure site in humans [40] although exposure to biomass fuel or motor vehicle exhaust elevated the abundance and alpha diversity of the lung microbiota in a rat model of exposure [41]. Depending on the exposure regimen or host species, different alterations of community patterns occur; these are a crucial factor in understanding the underlying pathogenesis of related diseases. Exposure to UFPs elevated cholesterol levels and reduced coprostanol levels in the cecum of mice [42]. Moreover, atherogenic lysophosphatidylcholine (18:1) and lysophosphatidic acid were found in the intestine and plasma of mice exposed to UFP. All these atherogenic lipids, including cholesterol, are potent mediators of pro-inflammatory responses, such as the recruitment of macrophages and neutrophils in the mucosa barrier. These lipids were negatively correlated with Actinobacteria, which was decreased by UFP exposure in a murine model [42]. Human epidemiological assessment in overweight and obese adolescents exposed to traffic-related air pollution also supported the negative correlation between changes in the microbiota and metabolic disorders [43]. Overall, the evidence in mice and humans provided a crucial insight into the contribution of PM-altered microbial communities to inflammatory cardiovascular diseases such as atherosclerosis. Moreover, PM-induced alteration of microbial communities can contribute to the metabolic activation of xenobiotic agents in PM [44]. In addition to effects on the community composition, PM-exposed gut microbiota displayed altered metabolic activities, which may affect the metabolism of endogenous biomolecules or the toxicity of PM-derived chemical components during mucosal exposure. Polycyclic aromatic hydrocarbons (PAHs) are among the most widespread organic pollutants generated by the incomplete combustion of fossil fuels and biomass. Although the parent PAH molecules are not estrogenic, in vitro evaluation in adult gut microbiota demonstrated the potent conversion of the parent PAHs to estrogenic hydroxyl metabolites, such as 1-OH pyrene and 7-OH benzo(a)pyrene [45]. In contrast, microbiota can protect against mutagen formation. For example, 2-nitrofluorene (NF), a representative nitro-PAH present in urban-air PM and diesel fuel emissions, can be reduced to 2-aminofluorene by the intestinal bacteria and is further acetylated to hydroxylated 2-acetylaminofluorene in the rat liver [46]. An alternate rat metabolism of NF results in the formation of mutagenic hydroxylated NF. However, mouse microflora tends to increase in 2-acetylaminofluorene, another DNA adduct from NF [46,47]. Therefore, depending on the host microbiota profile and the bioavailability of host cell metabolic enzymes, PM metabolites may be converted to either detrimental or inactive metabolites. Moreover, the PM-mediated alteration of the microbial community can determine the fate of PM-derived xenobiotics in human health and disease (Figure 2). 

### 2.4. Microbiota-Derived Uremic Solutes in Response to PM

The term uremic retention solutes (URS) refers to the components that accumulate in the blood and tissues during renal disease. Changes in the microbiota composition and community structure (dysbiosis) are associated with the production of 11 microbiota-derived uremic solutes [48,49]. Microbiota in normal conditions or dysbiosis can produce p-cresyl sulfate (PCS) from tyrosine, indoxyl sulfate (IS) from tryptophan, trimethylamine N-oxide from L-carnitine, dimethylglycine from choline, and glutarate from lysine [50]. Phenyl sulfate, cholate, hippurate, γ-guanidinobutyrate, 2-hydroxypentanoate, and phenaceturate are also considered URS. Moreover, the active metabolites in URS are formed by the combined actions of the microbial transformation and the host metabolic enzymes. For example, bacterial tryptophanase metabolizes dietary tryptophan to indole, which is subsequently hydroxylated to indoxyl by cytochrome P450 (CYP) isozyme 2E1 and finally sulfonated to indoxyl sulfate by sulfotransferases including SULT1A1 in the liver [51]. Therefore, hepatic dysfunction in patients with CKD and cirrhosis retards the formation of indoxyl sulfate and *p*-cresol sulfate due to impaired hepatic metabolism [52]. URS-induced renal injuries mostly result from inflammatory responses and radical production. Dysbiosis-induced indoxyl sulfate acts on the basolateral membrane of renal proximal tubular cells via binding to the organic anion transporter and causes inflammation and nephrotoxicity. PCS accumulates in kidney tubular cells, leading to the generation of ROS, proinflammatory cytokines, and hypoxia factors [53], which is consistent with patterns from the network analysis (Figure 1).

**Figure 3:**
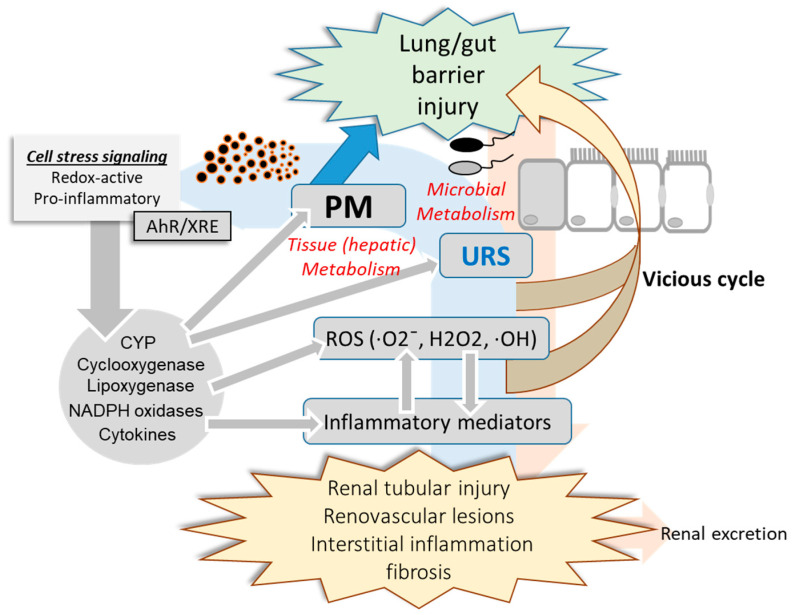
PM-induced alteration in mucosa-kidney axis. PM-insulted mucosal barrier allows the translocation of PM, uremic retention solutes (URS), and their metabolites which can have detrimental effects on the mucosa-kidney axis via stress signaling including AhR-linked pathways. Moreover, invasive microbes and harmful metabolites from the altered microbiota community can contribute to the systemic and renal inflammation during PM exposure. In addition to renal distress, reactive oxygen species (ROS), inflammatory mediators and the circulating xenobiotic agents including PM components and URS injure the mucosal barrier integrity in a feedback way.

As mentioned earlier, uremic toxins can be exposed to the circulatory system through the dysbiosis-disrupted leaky mucosal barrier [54]. Mechanistically, uremic toxins and urea-derived metabolites cause degradation of tight junction proteins [55,56], leading to increased translocation of luminal toxic metabolites to the vasculature and kidney. Moreover, mucosal microbiota may be involved in the regulation of xenobiotic metabolic enzymes and transporters in the liver [57,58]. Antibiotic-treated or germ-free animals show altered pharmacokinetics compared with intact hosts, which can be attributed to changes in CYP gene profiles [59]. Microbiota may regulate xenobiotic metabolic enzymes via microbial metabolites that can act as ligands for receptors involved in the induction of genes coding for xenobiotic metabolic enzymes or transporters [57,60]. Microbiota-derived uremic toxins can regulate the expression of genes coding for CYPs and inflammatory mediators via the aryl hydrocarbon receptor (AhR) [60]. Collectively, the PM-induced alterations in the microbiota community contribute to the production and metabolism of URS and PM in the mucosa, which can be translocated to the circulatory system via a disrupted mucosal barrier and has detrimental effects on the renovascular system (Figure 3). Circulating uremic metabolites can also modulate further metabolic and pharmacokinetic processes by regulating the expression and activities of host xenobiotic metabolic enzymes and transporters. Furthermore, PM, URS, and the pool of their metabolites may impact the mucosal barrier, forming a vicious feedback cycle.

### 2.5. Mechanistisms of Renal and Vascular Injuries in Response to PM

Circulatory PM and uremic metabolites are detrimental to the renal parenchymal and endothelial tissues in a similar way as the predicted network disruption in the mucosa (Figure 1). In particular, PM can generate ROS including hydroxyl radical (OH) mainly from transition metals and quinones in the airway mucosa [61]. In terms of molecular mechanism of toxicity,∙OH is one of strong genotoxic molecules that quickly bind to and injure DNA [62]. Furthermore, PM-derived redox-active compounds and oxidation products of the lipid membrane can serve as ligands for AhR via transcriptional activation of the xenobiotic responsive element (XRE), leading to expression of genes involved in diverse pathologic events in exposed cells [63]. As previously mentioned, URS and other active microbial metabolites also can contribute to the total pools of AhR ligands during PM exposure. In particular, AhR-XRE signaling mediates expression of ROS-producing metabolic enzymes including cyclooxygenase, lipoxygenase, CYP and NADPH oxidase [64,65,66,67,68,69]. In addition to effects on ROS production and xenobiotic metabolism, AhR-linked signaling is involved in proinflammatory cytokine production and cell death responses [70,71]. URS-induced oxidative stress and proinflammatory cytokines cause necrotic and apoptotic death of the renal tubular and renovascular cells [72,73]. Mechanistically, PM- or URS-activated AhR can disrupt the mitochondrial membrane potential or trigger other diverse cell death signaling pathways, which is the crucial step of renal tubular and renovascular tissue injuries during PM exposure [72,73,74,75]. In contrast, PM, URS, and their active metabolites attenuate the antioxidant capacity in response to the oxidative stress in the mucosa-renal axis. For example, phenyl sulfate, IS and PCS decrease glutathione level in the renal tubular cells [76]. Furthermore, URS-induced chronic distress facilitates progressive interstitial inflammation and renal fibrosis via tissue fibrotic factors including TGF-β1 and α-smooth muscle actin, which ultimately hasten CKD progression [77,78]. Taken together, PM and mucosa-derived metabolites cause renal tubular and endothelial injuries via the oxidative and proinflammatory stress signaling. Moreover, chronic inflammatory and fibrogenic processes aggravate the renal distress during PM exposure. 

## 3. Mucosal Interventions for PM-Induced Renal Injuries

### 3.1. Mucosa-Derived Endogenous Factors

The mucosal microbiota can function as an endocrine organ that metabolically influences vascular and renal physiology or disease progression by facilitating the production of metabolites, including short-chain fatty acids (SCFAs), as a result of carbohydrate and protein metabolism. SCFAs, such as acetate, propionate, and butyrate, may drive the release of enteroendocrine peptides such as serotonin and peptide YY in the GI tract [79,80,81]. Moreover, mice exposed to SCFAs experienced reduced ischemia-reperfusion kidney injury, which was associated with low levels of local and systemic inflammation, oxidative cellular stress, cell infiltration/activation, and apoptosis [82]. In contrast, animals exposed to PM or cigarette smoke had attenuated levels of SCFAs, and this effect persisted in their offspring [83,84]. Bacteroides, Bifidobacterium, Propionibacterium, Eubacterium, Lactobacillus, Clostridium, Roseburia, and Prevotella are the major bacteria related to the production of SCFAs [85], and PM can suppress SCFA production by directly altering these bacteria or indirectly inducing dysbiosis in the mucosal microbial community.

An epidemiological evaluation demonstrated that secretion of glucagon-like peptide-1 (GLP-1) from enteroendocrine cells was decreased by PM exposure [86]. Various types of nutrients, gut bacteria, and bacterial products are known to trigger the secretion of intestinal GLP-1 [87,88,89], which has a crucial role in counteracting the progress of metabolic diseases, including cardiovascular diseases and diabetes [89,90,91]. Therefore, PM-induced dysbiosis may alter GLP-1 production and its actions in the kidneys and vasculature. Although the distribution of GLP-1 receptors in the kidneys is a controversial topic, the GLP-1 receptor of the renal vasculature was confirmed to be involved in the beneficial action of GLP-1 in the kidneys [92]. In addition to glycemic control as the central action of GLP-1 in the pancreas, GLP-1 regulates glomerular filtration rate (GFR), but the mechanisms need to be clearly addressed [93]. Moreover, GLP-1 can control the inflammatory and oxidative stress occurring during metabolic renal disorders [94,95]. Collectively, gut-derived GLP-1 can counteract inflammatory and other pathologic outcomes in the gut-kidney axis that may be altered by PM exposure. 

### 3.2. Muco-Active Supplementation: Probiotics

Probiotic microbes can be beneficial by ingestion to improve the host renal health and integrity in response to internal or external insults [96]. As mentioned above, the microbial community is closely related to uremic toxin production and aggravates renal inflammation because the toxins are closely associate with renal cell toxicity [97]. Suppression of toxin production delayed the progression of renal failure. Therefore, the gut microbial community is considered a key target for intervention against mucosa-linked renal dysfunction. As a potent modifier of the distressed microbial community, probiotic agents containing Bifidobacteria and lactobacilli can elicit potential benefits in the management of CKD and uremic toxin-linked disease [98]. In addition to the popular probiotic application, probiotic supplements of *Bacillus pasteurii* and sporolactobacillus substantially decreased blood urea nitrogen levels and improved the life span of nephrectomized animals in the state of azotemia [99]. In another interventional study, Lebenin, a preparation consisting of antibiotic-resistant lactic acid bacteria, efficiently reduced the levels of indoxyl sulfate and p-cresol in patients [100]. However, the efficacy of probiotic application is highly variable depending on the treatment regimen and individual subject variation. In particular, as mucosal dominance of the aboriginal microbial groups can affect the colonization and maintenance of the alien probiotic species [101,102], more careful and systematic experimental settings in response to the host and microbiota features are needed to perform a convincing evaluation of the empiric probiotic treatment.

### 3.3. Muco-Active Supplementation: Prebiotics and Other Agent-Based Interventions

Prebiotics are non-digestible food ingredients that stimulate the growth and/or activity of commensal microbes in the gastrointestinal tract [1]. Prebiotics help the activity of probiotics as prey of beneficial bacteria. Similar to the actions of probiotics, prebiotics, including inulin, galacto-oligosaccharides, fructo-oligosaccharides, xylitol, lactulose, and lactitol, also alter the microbial community. Moreover, synbiotic treatment induces synergetic effects beyond the probiotics’ beneficial actions, such as inhibiting harmful bacteria in the intestines and increasing the beneficial bacterial community. In one clinical evaluation, patients receiving hemolysis who had high concentrations of PCS were treated with prebiotic oligofructose-enriched inulin (p-inulin) [103]. After p-inulin treatment, although there was no difference in indoxyl sulfate, there was a marked difference in PCS levels. The remission in the generation rate and serum concentration of PCS was significantly associated with prebiotic intervention. As mentioned previously, synbiotic intervention was also verified to be effective in uremic syndromes in a clinical trial [104]. Synbiotic treatment with *Lactobacillus casei* strain Shirota and *Bifidobacterium breve* strain Yakult as probiotics and galacto-oligosaccharides as a prebiotic efficiently attenuated serum p-cresol levels in patients with end-stage renal disease [104]. In addition to the consideration of the host and microbiota features in the probiotic treatment, an optimized combination of synbiotic intervention is crucial to achieve proper therapeutic efficacy. 

From the perspective of the microbial community, diet is another deterministic factor that improves mucosa-associated renal distress [105]. Among various dietary interventions, omega-3 fatty acid-rich fish oil and soybean-based diet ameliorated inflammation (reduced CRP levels) [106,107]. In addition, diets containing vitamin D and heparin can be used to reduce the inflammatory responses in inflammatory diseases by reducing pro-inflammatory cytokines and increasing anti-inflammatory cytokines. Uremic solutes activated the inflammatory response in monocytes, which was counteracted by 25-vitamin D supplementation [108]. Therefore, the combination of dietary application of anti-inflammatory intervention and a microbiota modifier may be a promising strategy to efficiently mitigate the clinical outcomes.

### 3.4. Binders of Uremic Toxins (Sorbents)

AST-120 is a well-known absorbent that can absorb indoxyl sulfates. AST-120 is an oral intestinal spherical carbon absorbent consisting of porous carbon particles insoluble in water or common organic solvents. An experimental study using an animal model to assess the effects of AST-120 showed that rats treated with AST-120 had decreased levels of indoxyl sulfate in both the serum and urine and reduced expression of profibrotic genes, such as TGF-β1, ultimately slowing chronic renal failure [109]. In prospective clinical trials of AST-120 in patients with CKD, the rate of decline in GFR was substantially attenuated by AST-120 treatment [110]. Moreover, AST-120 retarded CKD progression by delaying dialysis initiation in patients with CKD, proving a potential intervention against chronic renal failure [111]. Another potent binder of uremic toxins is sevelamer hydrochloride (SH), a non-calcium, non-aluminum phosphate binder [37]. In addition to its phosphate binding ability, SH has the ability to bind uremic toxins. One clinical study showed that SH markedly reduced serum levels of phosphate and PCS in patients undergoing hemodialysis [37]. Although SH can be a useful therapeutic agent to bind uremic toxins in patients with uremic disorders, additional mechanistic and translational evaluations are warranted in various phases of renal dysfunction to explore the associated complications and allow safer clinical application.

## 4. Conclusions

Early impacted mucosal linings in the lungs and gut are crucial barriers to the systemic translocation of PM and development of extrapulmonary disorders, including kidney disease, from environmental insult as summarized in the Table 1. In addition to the disruption of barrier integrity by mucosa-deposited PM, toxic stress can stimulate production of inflammatory mediators. These mucosal events are also associated with changes in the mucosal microbial community, which can intrude into the circulation and induce a proinflammatory attack on renal tissues. Moreover, altered microbial metabolites of nutrients, including uremic retention solutes, are involved in renal disease progression, which are also detrimental to the mucosal barrier in a vicious feedback pathway. Both mucosal microbiota and nutrients play pivotal roles in mediating the individual susceptibility to environment-associated distress. In contrast, mucosa-associated renal disease can be counteracted by microbial metabolites, such as SCFAs, and host cell-derived endogenous protective factors, including incretins and protective enteroendocrine peptides. Moreover, probiotic and prebiotic applications target the microbial community and host immunity.

Personalized modulation of mucosal communication among mucosal cells, microbiota, nutrients, and other xenobiotics would be a promising intervention against persistent environmental insults.

## Figures and Tables

**Table 1:** Mucosal etiologies and interventions in response to PM exposure.

	Mucosal Etiologies	Mucosal Interventions
	Detrimental microbial community	Probiotic, Prebiotic, and other community modulators (SCFA, GLP-1)
Input	Redox-active compounds (reactive oxygen species, oxidation end products of lipid and proteins)	Endogenous and dietary antioxidants
	uremic retention solutes (nutritional and microbial metabolites)	Uremic toxin binders
	Proinflammatory mediators	Anti-inflammatory agents, microbial and endogenous regulators (SCFA, GLP-1)
Outcomes	Barrier disruption Renal injury, inflammation, fibrosis	Maintenance of barrier integrity Limited exposure of renal tissues to mucosa-derived insults

## Abbreviations

2-nitrofluorene, NF; aryl hydrocarbon receptor, AhR; chronic kidney disease, CKD; chronic obstructive pulmonary disease, COPD; cytochrome P450, CYP; glomerular filtration rate, GFR; glucagon-like peptide-1, GLP-1; indoxyl sulfate, IS; Kyoto Encyclopedia of Genes and Genomes, KEGG; Particulate matter, PM; p-cresyl sulfate, PCS; Polycyclic aromatic hydrocarbons, PAHs; reactive oxygen species, ROS; short-chain fatty acids, SCFAs; type 2 diabetes, T2D; ultrafine particulates, UFP; uremic retention solutes, URS.
